# *In vitro* synthesis of linear α-1,3-glucan and chemical modification to ester derivatives exhibiting outstanding thermal properties

**DOI:** 10.1038/srep30479

**Published:** 2016-07-29

**Authors:** Sakarin Puanglek, Satoshi Kimura, Yukiko Enomoto-Rogers, Taizo Kabe, Makoto Yoshida, Masahisa Wada, Tadahisa Iwata

**Affiliations:** 1 Department of Biomaterial Sciences, Graduate School of Agricultural and Life Sciences, The University of Tokyo, 1-1-1 Yayoi, Bunkyo-ku, Tokyo 113-8657, Japan; 2 Materials Structure Group 1, Research & Utilization Division, Japan Synchrotron Radiation Research Institute (JASRI), 1-1-1 Kouto, Sayo-cho, Sayo-gun, Hyogo 679-5198, Japan; 3 Department of Environmental and Natural Resource Sciences, Tokyo University of Agriculture and Technology, 3-8-1 Harumi-cho, Fuchu-shi, Tokyo, 183-8509, Japan; 4 Department of Forest and Biomaterials Science, Graduate School of Agriculture, Kyoto University, Kitashirakawa Oiwake-cho, Sakyo-ku, Kyoto 60608502, Japan; 5 Department of Plant & Environmental New Resources, College of Life Sciences, Kyung Hee University, 1, Seocheon-dong, Giheung-ku, Yongin-si, Gyeonggi-do, 446-701, Korea

## Abstract

Bio-based polymer is considered as one of potentially renewable materials to reduce the consumption of petroleum resources. We report herein on the one-pot synthesis and development of unnatural-type bio-based polysaccharide, α-1,3-glucan. The synthesis can be achieved by *in vitro* enzymatic polymerization with GtfJ enzyme, one type of glucosyltransferase, cloned from *Streptococcus salivarius* ATCC 25975 utilizing sucrose, a renewable feedstock, as a glucose monomer source, via environmentally friendly one-pot water-based reaction. The structure of α-1,3-glucan is completely linear without branches with weight-average molecular weight (M_w_) of 700 kDa. Furthermore, acetate and propionate esters of α-1,3-glucan were synthesized and characterized. Interestingly, α-1,3-glucan acetate showed a comparatively high melting temperature at 339 °C, higher than that of commercially available thermoplastics such as PET (265 °C) and Nylon 6 (220 °C). Thus, the discovery of crystalline α-1,3-glucan esters without branches with high thermal stability and melting temperature opens the gate for further researches in the application of thermoplastic materials.

Polysaccharides, natural polymers composed of sugar units linked via glycosidic bonds, have been considered as interesting bio-based materials for utilization in many applications such as plastics and biomedical field with currently increasing amount of researches. The advantages are that they are made from renewable resources supporting the trend to reduce the consumption of plastics made from petrochemicals and with the concept of carbon neutrality they can be regarded as eco-friendly materials^1^. Plants typically produce polysaccharides such as well-known cellulose and hemicelluloses such as xylan and glucomannan. However, hemicelluloses are branched and they are extracted from wood via acid process leading to chain degradation that lowers the molecular weight. Besides, microorganisms can synthesize many polysaccharides as well such as pullulan from *Aureobasidium pullulans*, curdlan from *Agrobacterium* spp.; *Rhizobium* spp.; *Cellulomonas* spp., dextran from *Leuconostoc* spp.; *Streptococcus* spp., and hyaluronic acid from *Streptococcus* spp.; *Pasteurella multocida*^2^.

The α-1,3-glucan, a water insoluble polysaccharide produced by microorganisms, is found primarily in the cell wall of several nonpathogenic and pathogenic fungi such as *Penicillium* spp., *Eupenicillium* spp., and *Aspergillus* spp^3^,^4^,^5^,^6^. In addition, α-1,3-glucan is also found as an extracellular polysaccharide produced by *Streptococcus* spp. present in the oral cavity, enhancing the formation of dental plaque and the induction of dental caries^7^. In the latter case, glucosyltransferases (Gtfs) or commonly named glucansucrases, enzymes produced by lactic acid bacteria, catalyze the extracellular synthesis of glucan^8^. Gtfs enzymes, which produce dental plaque glucans, can be classified by 2 criteria: the water solubility behavior of produced glucan and the requirement of primer dextran for enhancing the reactivity^9^. Each type of Gtfs enzymes produced dental plaque glucans with different composition of α-1,6- and α-1,3-linkages, which contribute the water-soluble and -insoluble characteristics to the materials respectively^8^.

The study of enzymes and gene encoding enzymes was revealed since more than 25 years ago in order to develop the preventive strategy against dental caries^10^,^11^,^12^,^13^. However, only few studies on the application of α-1,3-glucan from different resources have been reported up to now despite that it might be a potentially low-cost polymer, which could be developed for new bio-based materials. One application study recently revealed fibrinolytic, anti-inflammatory and anti-microbial properties of α-1–3-glucan produced from *Streptococcus mutans* MTCC 497 after chemical modification to sulfated derivative^14^ and another one showed anti-tumor activities of phosphated α-1–3-glucan from *Poria cocos* mycelia^15^.

Herein, water insoluble glucan-synthesizing glucosyltransferase, designated as GtfJ, from *Streptococcus salivarius* ATCC 25975 as an enzyme production scheme shown in Fig. 1a, the dominant bacteria species on the dorsum of the tongue in human, has been being used for *in vitro* enzymatic polymerization of α-1,3-glucan because it can produce the glucan composed mainly of α-1,3-linkage^9^. *In vitro* enzymatic polymerization of natural polysaccharides has been reported in many published papers, but the main obstacle is that the monomers need to be designed and modified with complicated procedures^16^,^17^,^18^. With this enzyme, sucrose can be directly used as a starting material without modification steps, and the glucan production is started from the enzymatic cleavage of sucrose into glucose and fructose, and then glucosyl residues are transferred to a growing glucan chain as synthesis scheme shown in Fig. 1b^19^. According to Simpson *et al.*^9^, the presence of small amount of α-1,6-linkage in the glucan produced by GtfJ might be occurred by the addition of primer dextran, α-1,6-glucan, during the glucan production. Therefore, interestingly, the reaction in the absence of primer dextran, would make the linear α-1,3-glucan without branches and lead to superior properties of materials.

Nevertheless, thermoplasticity of polysaccharides is the limitation for material utilization. Up until now, many researchers have been attempting to improve this drawback by various techniques. One interesting method is the introduction of acyl groups to the hydroxyl groups of sugar units, or esterification, which raised the thermoplastic properties of cellulose known as so-called cellulose acetate, and also improved the thermal properties and solubility of starch, xylan, glucomannan and curdlan in reported researches recently^20^,^21^,^22^,^23^.

Here we report the preparation of α-1,3-glucan by *in vitro* enzymatic polymerization with GtfJ enzyme without the addition of primer dextran and its linear structure was confirmed by NMR experiments. Homogeneous esterification of α-1,3-glucan was then employed, for the first time, aiming to synthesize ester series with variation in alkyl chain length. In addition, from a plastic application perspective, the functional properties of α-1,3-glucan esters such mass, molecular weight distribution, and thermal properties were explored in greater detail in comparison with other polysaccharides and commercially available products.

## Results

### *In vitro* synthesis of linear α-1,3-glucan

**Figure 1:**
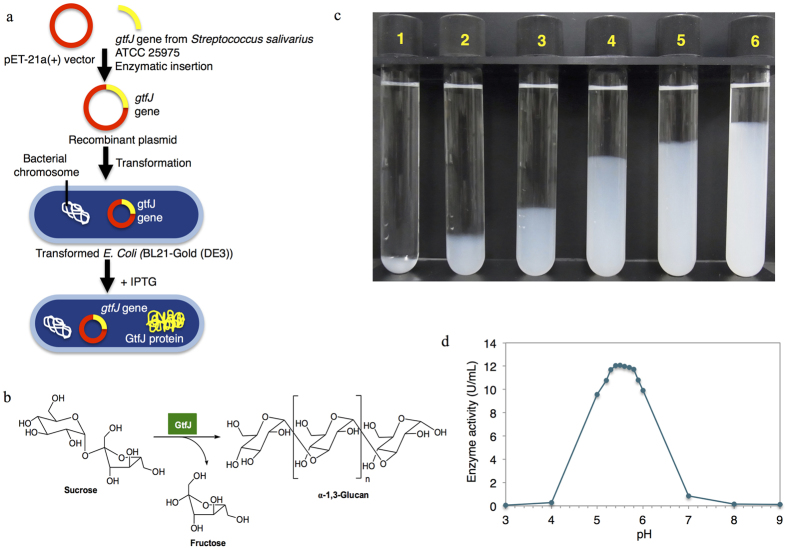
Synthesis of α-1,3-glucan. (**a)** The source of GtfJ enzyme. IPTG is isopropyl β-D-1-thiogalactopyranoside, a protein inducer. (**b**) Scheme of enzymatic polymerization of α-1,3-glucan. (**c)** Pictures of enzymatic polymerization of α-1,3-glucan produced at 30 °C. The number indicates the polymerization day. (**d**) Effect of pH on GtfJ activity at 30 °C. The enzyme activity of GtfJ is defined as the amount of released fructose per minute (μmole/min, U) per one mL of GtfJ at the initial state of reaction.

Firstly, GtfJ enzyme was produced by culturing *Escherichia coli (E. coli)* expressing GtfJ cloned from *Streptococcus salivarius* ATCC 25975, and then extracting and purifying the synthesized enzyme from the culture medium. The acid-base condition of reaction with GtfJ is a significant factor for effective production of α-1,3-glucan. In order to find optimum pH of reaction with GtfJ enzyme, the sucrose solutions containing 100 mM citrate buffer at different values of pH ranging from 3 to 9 were incubated at 30 °C. The GtfJ expressed high activity at pH of 5–6, meanwhile its activity decreased dramatically at pH below 5 and above 6. The optimum pH for the enzymatic reaction was 5.3–5.8 as shown in Fig. 1d. Thus, GtfJ works effectively in weak acidic region of pH and this phenomenon is similar to other different Gtfs from *Streptococcus mutans* 6715, *Aureobasidium* and *Pseudomonas mesoacidophila* MX-45^24^,^25^,^26^,^27^,^28^. Consequently, enzymatic reaction at pH 5.5 was performed throughout this research.

Enzymatic polymerization converting sucrose to α-1,3-glucan was simply done via one-pot water-based synthesis, contacting sucrose solution with GtfJ enzyme. At reaction temperature of 30 °C, the sucrose solution containing GtfJ was colorless at the beginning. After that, the mixture became turbid suggesting the formation of water-insoluble glucan. The appearance of the solution changed from clear to cloudy as reaction time increased, the synthesized glucan dispersed as suspended white solid in water medium. As shown in Fig. 1c, the amount of white solids increased over reaction time.

**Figure 2:**
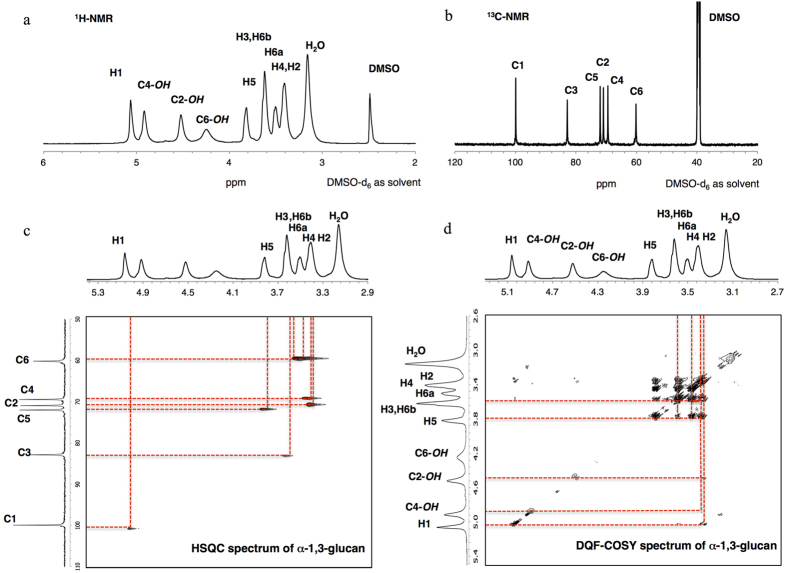
One- and two- dimensional NMR spectra of α-1,3-glucan. (**a)** ^1^H-NMR spectrum of α-1,3-glucan. (**b)** ^13^C-NMR spectrum of α-1,3-glucan. (**c**) HSQC NMR spectrum of α-1,3-glucan. (**d**) DQF-COSY NMR spectrum of α-1,3-glucan. All samples were prepared in DMSO-d6.

1D ^1^H- and ^13^C-NMR and 2D ^1^H-NMR (DQF-COSY) and ^1^H-^13^C NMR (HSQC) experiments were carried out to confirm and elucidate the structure of water-insoluble glucan and the results are displayed in Fig. 2. The synthesized glucans were insoluble in chloroform but soluble in DMSO, thus the solution of each sample for NMR measurement was prepared in DMSO-d6. ^13^C-NMR spectrum of this glucan in Fig. 2b displayed six major peaks at chemical shift of 60.2, 69.5, 70.9, 72.0, 82.9 and 99.9 ppm and these were assigned to C-6, C-4, C-2, C-5, C-3 and C-1 respectively. These chemical shifts are consistent with those from ^13^C-NMR spectra of α-1,3-glucan isolated from *Penicillium chrysogenum*, *Aspergillus wentii* and *Ramaria botrytis*^5^,^6^,^24^. ^13^C signals of glucose unit with α-1,6-linkage were not present in the spectra of water-insoluble glucan produced in this research due to the absence of primer dextran molecules, which contribute α-1,6-glycosidic bond to the glucan, in comparison with insoluble glucan obtained from sucrose solution containing GtfJ with a presence of dextran T-10as primer^9^. This result confirms that glucose units of synthesized water-insoluble glucan consist of only α-1,3-linkage, thus the synthesized glucan was linear α-1,3-glucan without branching structure.

^1^H-NMR spectra of α-1,3-glucan prepared from enzymatic polymerization with GtfJ enzyme were presented in past research papers but the elucidation of ring ^1^H was not yet clearly shown and those α-1,3-glucan reported in related published papers contained branching α-1,6-linkage^9^,^25^. Herein, the HSQC spectrum as in Fig. 2c, which gives the information of correlation between ring ^1^H and ^13^C, enhanced the elucidation of the ^1^H peaks of α-1,3-glucan, and thus the chemical shifts at 3.41, 3.51, 3.62, 3.82 and 5.06 were assigned to H-2 and H-4, H-6a, H-3 and H-6b, H-5, and H-1 respectively. The ^1^H-NMR peaks of H-2 and H-4 were not clearly separated, but from HSQC spectrum the peak of H-4 was at slightly higher chemical shift than that of H-2. However, the chemical shifts at 4.25, 4.52 and 4.92 were still unable to be assigned by only HSQC spectrum. Then, DQF-COSY, which shows the correlation between nearby ^1^H, was performed. The result revealed that chemical shifts at 4.52 and 4.92 belong to ^1^H of hydroxyl group at C-2 and C-4 respectively as shown in Fig. 2d. The chemical shift at 4.25 should be assigned to C-6, but no correlation peak appeared, and hence fully assigned ^1^H-NMR spectrum is shown in Fig. 2a. The ^1^H signals of hydroxyl groups were observable in this case because strong hydrogen bonds with DMSO-d6, solvent for NMR measurement, slow down the intermolecular proton exchange, leading to the appearance of ^1^H signals from hydroxyl group.

**Figure 3:**
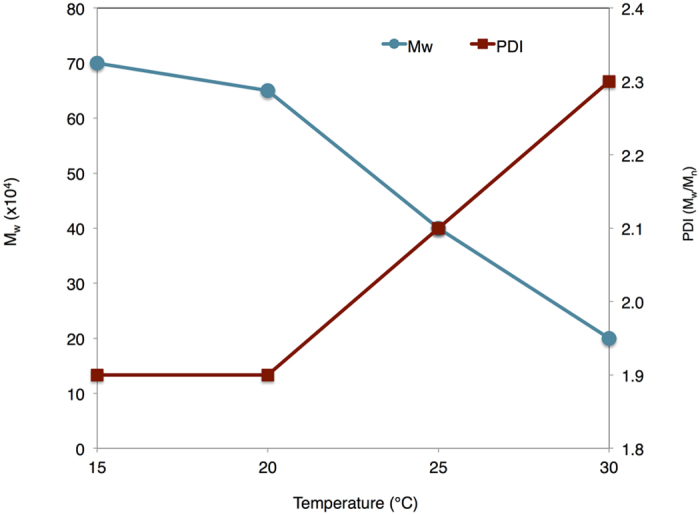
Effect of reaction temperature on molecular weight and molecular weight distribution of α-1,3-glucan. M_w_ is the weight-average molecular weight. PDI is polydispersity index calculated as the weight-average molecular weight (M_w_) divided by the number-average molecular weight (M_n_).

In addition, average molecular weight and yield of α-1,3-glucan produced at different temperature for 2 weeks of reaction time were investigated. As shown in Fig. 3, the M_w_ of α-1,3-glucan increased and polydispersity index (PDI) decreased with a reduction of reaction temperature from 30 to 15 °C, in the mean time, however, the yield decreased when reaction temperature was reduced. M_w_ of α-1,3-glucan produced at 30 °C were 2.0 × 10^5^, whereas, interestingly, M_w_ reached 7.0 × 10^5^ at reaction temperature of 15 °C.

### Synthesis of α-1,3-glucan esters

Acetate and propionate ester derivatives of α-1,3-glucan were synthesized through homogeneous method. In order to restrain the excessive chain degradation of α-1,3-glucan due to acid condition during esterification, acetic or propionic anhydride was used throughout the synthesis and pyridine, which can neutralize the acid, was selected as a catalyst^29^. The synthesized α-1,3-glucan esters were soluble in CHCl_3_. The peaks of methyl and methylene groups and ring protons of α-1,3-glucan esters were assigned by 1D ^1^H- and ^13^C-NMR and 2D ^1^H-NMR (DQF-COSY) and ^1^H-^13^C NMR (HSQC) experiments and the assigned ^1^H peaks were clearly reported in method section and supplementary information. Results of esterification of α-1,3-glucan by acid anhydrides are shown in Table 1. DS indicates the numbers of carboxylate group substituting at hydroxyl groups of glucose unit of α-1,3-glucan. Both acetate and propionate esters have DS of 3, implying that esterification of α-1,3-glucan was completely done via this method. M_w_ of α-1,3-glucan acetate and propionate were 1.6 × 10^5^ and 2.1 × 10^5^ respectively, and both esters have M_w_/M_n_ value of 2.3. Mw of α-1,3-glucan esters were less than that of neat α-1,3-glucan to some extent. In addition, the yield of α-1,3-glucan acetate and propionate were 73 and 72% respectively, comparatively high yield.

### Material characterization

**Figure 4:**
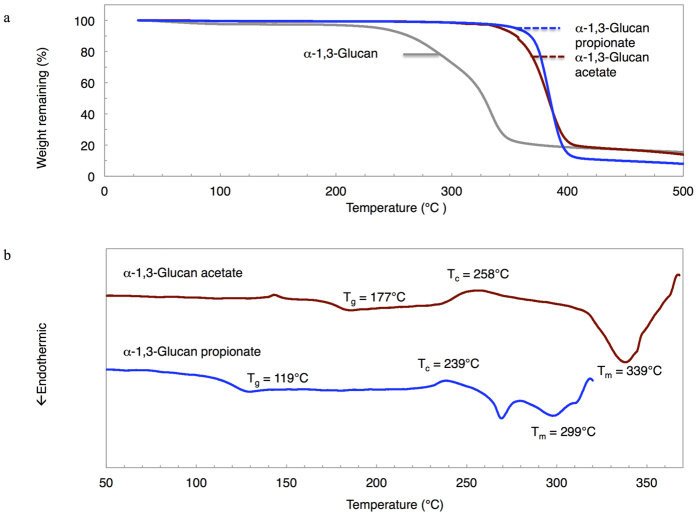
Thermal characterizations of α-1,3-glucan esters. (**a)** TGA curves of α-1,3-glucan (grey line), α-1,3-glucan acetate (dark red line) and α-1,3-glucan propionate (blue line). (**b**) DSC curves of α-1,3-glucan acetate (dark red line) and α-1,3-glucan propionate (blue line). All DSC curves are from 2^nd^ run.

Basically, thermal properties are important for considering application and establishing processing condition of materials. Figure 4a shows the thermal degradation behavior of α-1,3-glucan esters from 30 to 500 °C. The degradation temperature at 5% weight loss (T_d.5%_) deduced from TGA curves as shown in Table 1 represents the stability of α-1,3-glucan esters over temperature as a criteria for consideration in processing condition. T_d.5%_ of α-1,3-glucan esters were ca. 350 °C, approximately 120 °C higher than that of neat α-1,3-glucan, thus the introduction of ester groups in place of hydroxyl groups in glucose units enhances the thermal stability of materials. This improvement in decomposition temperature was also noticed in ester series of starch, glucomannan, xylan and curdlan^20^,^21^,^22^,^23^. In addition, evaporation of water was shown in the TGA curve of α-1,3-glucan from 30 °C to around 100 °C. It can be explained that water was adsorbed in α-1,3-glucan, so α-1,3-glucan should be dried well before the esterification.

Thermal transition behavior of α-1,3-glucan esters was analyzed with DSC. Figure 4b shows 2^nd^ run DSC curves of both esters. The endothermic melting peak appeared in both α-1,3-glucan esters. This characteristic was also remarked in curdlan esters reported in the previous article that the melting endotherm appeared in both acetate and propionate esters^23^. In addition, intriguingly, the melting temperatures (T_m_) of α-1,3-glucan acetate and propionate were 339 and 299 °C respectively, comparatively higher than commercially available polymers. These T_m_ values of α-1,3-glucan acetate and propionate were higher than T_m_ of commercially available semicrystalline polymers such as polyethylene terephthalate (245–265 °C)^30^, bio-based polylactic acid (130–180 °C)^30^, cellulose acetate with DS of 2.92 (293 °C)^31^, and also curdlan acetate and propionate (287 and 213 °C respectively)^23^ from previous research which are captivating our attention currently. Furthermore, the glass transition temperature (T_g_) were detectable in both α-1,3-glucan acetate and propionate, and these values were 177 and 119 °C respectively. In addition, both T_g_ and T_m_ of α-1,3-glucan esters were dependent on the chain length of carboxylate group i.e. their values were inversely proportional to the carbon number of carboxylate, T_g_ and T_m_ of α-1,3-glucan acetate are higher than those of propionate. Consequently, α-1,3-glucan acetate and propionate, which possess high T_m_ and T_g_, are particularly interesting and further investigation in their crystal structures is required to gain a deeper understanding in properties of materials.

## Discussion

The extracted GtfJ enzyme from recombinant *E. coli* can effectively catalyze the one-pot water-based enzymatic polymerization of linear α-1,3-glucan without branches.

The optimum pH for the catalysis of this enzyme was 5.3–5.8. The synthesis process is environmentally friendly with a reaction in water medium, without organic solvent, and also convenient, only mixing sucrose solution with enzyme and storing at designated temperature. The raw material, sucrose as a source of monomer, is also from renewable resources and commercially available. Thus, α-1,3-glucan can be considered as a potentially low-cost polymer for future prospect. In addition, reaction temperature affected M_w_ and yield of α-1,3-glucan i.e. lower temperature led to higher molecular weight and M_w_ increased approximately 3.5 times with a reduction of reaction temperature from 30 to 15 °C. At lower reaction temperature, the active chains from the chain initiation are generated at lower amount than those generated at higher temperature, thus higher molecular weight of polymers can be obtained. As a result, the molecular weight of α-1,3-glucan can be adjusted by reaction temperature, hence the molecular weight and yield of α-1,3-glucan are the significant factors to be developed in further researches.

**Figure 5:**
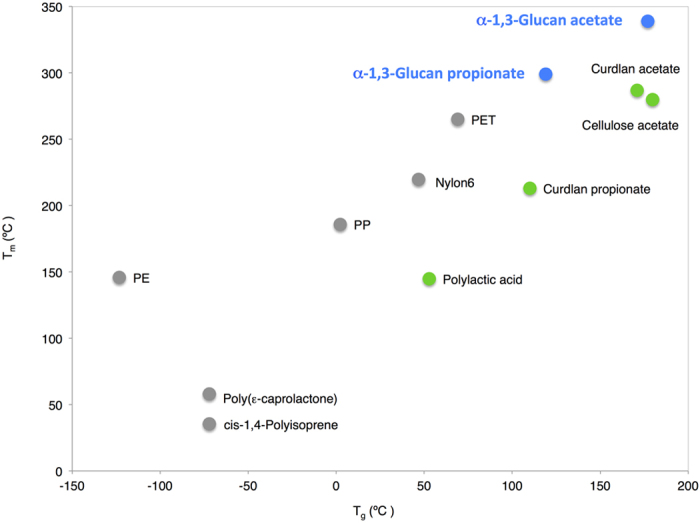
Comparison of glass transition and melting temperature between those of α-1,3-glucan acetate and propionate, esters of other polysaccharides and commercially available polymers. T_g_ and T_m_ are glass transition and melting temperature respectively. PE, PP and PET are polyethylene, polypropylene and polyethylene terephthalate respectively.

With the aim to improve this polysaccharide, ester series of α-1,3-glucan were synthesized via the homogeneous method with acid anhydride and pyridine in LiCl/DMAc, and then fully substituted (DS = 3) products were successfully obtained. Thermal degradation temperature was improved after esterification from 237 °C to ca. 350 °C because of the replacement of hydroxyl groups by acyl groups. DSC curves suggested a presence of crystal structure in α-1,3-glucan esters. T_m_ was detected in both α-1,3-glucan acetate and propionate, and an acetate ester shows higher T_m_ as a result of lower carbon chain length of acyl group. Currently, the crystal structures of these materials are now being investigated with X-ray experiments. Figure 5^23^,^32^ shows the superior melting and glass transition temperature of α-1,3-glucan acetate and propionate over commercially available petroleum-based thermoplastics and currently interesting bio-based polymers, thus these materials are regarded as promising candidates for future thermoplastic application.

In conclusion, this research shows that α-1,3-glucan can be conveniently produced with many advantages such as low-cost materials and reaction in water medium, and crystalline α-1,3-glucan esters with outstanding thermal properties - high thermal stability and melting temperature - are of interest for developing new thermoplastic materials.

## Methods

### Preparation of GtfJ Enzyme

*Escherichia coli (*BL21-Gold (DE3) (Stratagene, USA) and vector pET-21a(+) (Novagen, USA)) expressing GtfJ cloned from *Streptococcus salivarius* ATCC 25975 were grown in 1 L of Luria-Bertani (LB) medium supplemented with 100 μg/mL of ampicillin (Wako Pure Chemical Industries) and 0.5 mM isopropyl β-D-1-thiogalactopyranoside (IPTG, Wako Pure Chemical Industries) as an antibiotic and a protein inducer respectively. The cells were harvested by centrifugation and then resuspended in 40 mL of lysis buffer (5 mM imidazole, 20 mM tris-HCl buffer pH 8.0, 300 mM NaCl, 0.5 mg/mL lysozyme, 1 mM dithiothreitol and one tablet of protease inhibitor (cOmplete™, EDTA-free)). Subsequently, the cells were sonicated for 10 min. Unbroken cells and cell debris were removed by centrifugation. The supernatant from the previous cell lysis was purified at 4 °C by immobilized metal affinity chromatography technique using nickel-charged nitrilotriacetic acid (Ni-NTA) column (Bio-Scale™, Bio-rad). Elution was carried out with 200 mM imidazole buffer solution (200 mM imidazole, 20 mM tris-HCl buffer pH 8.0 and 300 mM NaCl) at a flow rate of 1 mL/min and fractions of 5 ml each were collected.

The GtfJ protein was identified by polyacrylamide gel electrophoresis. A 15 μL of each sample was prepared by mixing with 15 μL of electrophoresis sample buffer containing bromophenol blue and then heated at 95 °C for 5 min. The prepared samples were directly applied onto the separation gel and the electrophoresis was carried out at 30 μA for 90 min. After gel electrophoresis, the sample fractions containing GtfJ were combined together and then precipitated with (NH_4_)_2_SO_4_ at 70% saturation followed by centrifugation. The precipitated enzyme was resuspended with 5 mL of 5 mM tris-HCl buffer pH 7.2 and finally desalting was carried out with PD-10 desalting column (Sephadex^®^ G-25 medium, GE healthcare).

The GtfJ activity was measured in term of the fructose amount produced from the hydrolysis of sucrose. It was measured by UV-Vis spectrophotometry at 340 nm with enzyme kits (D-glucose/D-fructose UV-test, R Biopharm AG, Darmstadt, Germany). One unit of GtfJ is defined as the amount of enzyme that can catalyze the conversion of one μmole sucrose to reducing sugar (fructose) in a minute at the initial state of reaction, first 30 minutes. The reactions were employed under the following condition: 50 mM sucrose solution, 100 mM citrate buffer, and enzyme 1/10 of total reaction volume. For the study of effect of pH on enzyme activity, the pH of 100 mM citrate buffer was varied from 3–9.

### *In vitro* synthesis of α-1,3-glucan

1 M Sucrose solution containing 0.1% NaN_3_ and 100 mM citrate buffer (pH 5.5) was prepared into 4 portions of 1 L each. After adding extracted GtfJ with 0.01 U/mL, each portion was incubated at 30 °C, 25 °C, 20 °C and 15 °C for 2 weeks (until no more fructose produced). The resulting insoluble material was collected by centrifugation and resuspended with DI water 5 times. Finally, the obtained samples were vacuum freeze dried overnight.

### Synthesis of α-1,3-glucan esters

Dried α-1,3-glucan with MW of 2.0 × 10^5^ (0.5 g), dried LiCl (0.45 g) and DMAc (10 mL) were mixed and stirred at 70 °C for 1 h (until the solution turned clear). The temperature was reduced to 60 °C and then 2 mL of pyridine was added into the solution followed by the dropwise addition of 2 mL of acid anhydride (Wako Pure Chemical Industries). The solution was then continually stirred at 60 °C for 48 h (acetic anhydride) and 72 h (propionic anhydride). After the reaction, the solution was cooled down to the room temperature and then precipitated in methanol. The precipitate was filtered, washed with methanol and deionized water, and dried in vacuo.

^1^H-NMR spectrum was used to calculate the degree of substitution (DS) of carboxylate groups for hydroxyl groups in the glucose unit of α-1,3-glucan esters from the ratio of peak area of methyl protons ([CH_3_]) in carboxylate group to that of ring protons in the glucose unit ([ring-H]), DS = ([CH_3_]/3)/([ring-H]/7).

#### α-1,3-Glucan acetate

^1^H NMR (500 MHz, CDCl_3_, δ, ppm): 2.10 (-CH_3_), 4.07 (H-3, H-5, H-6), 4.65 (H-2), 5.05 (H-4), 5.20 (H-1).

#### α-1,3-Glucan propionate

^1^H NMR (500 MHz, CDCl_3_, δ, ppm): 1.10 (-CH_3_), 2.36 (-CH_2_-), 4.05 (H-3, H-5, H-6), 4.59 (H-2), 5.02 (H-4), 5.20 (H-1).

### Sample characterization

Nuclear magnetic resonance (NMR): ^1^H, ^13^C, DQF-COSY (double quantum-filtered correlation spectroscopy), and HSQC (heteronuclear single-quantum correlation) NMR spectra were measured with JEOL NMR spectrometer operating at 500 MHz and 25 °C. Samples of α-1,3-glucan for NMR analysis were prepared by dissolving 15 mg of a sample in 1 mL of DMSO-d6. In the case of α-1,3-glucan esters, CDCl_3_ was used as solvent with the same concentration as α-1,3-glucan. Chemical shifts (δ in ppm) were recorded in relative to the resonance of tetramethylsilane (TMS; δ = 0) in the case of CDCl_3_ and to the resonance of an internal solvent in the case of DMSO-d6.

Gel permeation chromatography (GPC): Number-average (M_n_) and weight- average (M_w_) molecular weights and polydispersity indices (M_w_/M_n_) were measured with GPC system composed of a CBM-20 A communications bus module, a DGU-20A3 degasser, an LC-6AD liquid chromatograph, an SIL-20AC HT auto sampler, an RID-10 A refractive index detector, and a CTO-20A column oven (Shimadzu Corp.). In the case of α-1,3-glucan, 20 mM LiCl in DMAc was used as an eluent with the flow rate of 0.6 mL/min and the column oven was set at 40 °C. CHCl_3_ was used as the eluent for α-1,3-glucan esters with the same condition. Polystyrene standards (Shodex^®^ Standard SM-105, Showa Denko K.K.) and polyethylene glycol/poly ethylene oxide standards (Sigma-Aldrich) were used to calibrate the molecular weights of α-1,3-glucan esters and α-1,3-glucan respectively.

Thermogravimetric analysis (TGA): Thermal decomposition of samples was observed with PerkinElmer STA 6000 under nitrogen gas environment. The thermogram was scanned from 30 °C to 600 °C at heating rate of 10 °C/min.

Differential scanning calorimetry (DSC): 2 mg of sample was filled in aluminum pan for analysis of glass transition, crystallization and melting temperature with PerkinElmer DSC 8500 under nitrogen gas atmosphere. The sample was first heated from 0 °C to 380 °C and 330 °C for α-1,3-glucan acetate and α-1,3-glucan propionate respectively at the heating rate of 100 °C/min, then cooled down to −30 °C at 100 °C/min and second heated to the same temperature with the same heating rate as first heating.

## Additional Information

**How to cite this article**: Puanglek, S. *et al.* *In vitro* synthesis of linear α-1,3-glucan and chemical modification to ester derivatives exhibiting outstanding thermal properties. *Sci. Rep.* **6**, 30479; doi: 10.1038/srep30479 (2016).

## Supplementary Material

Supplementary Information

## Figures and Tables

**Table 1:** Synthesis of α-1,3-glucan esters.

Sample	DS	Reaction Time (hours)	M_w_(x10^4^)	M_n_(x10^4^)	M_w_/M_n_	T_d.5%_(°C)	T_g_(°C)	T_c_(°C)	T_m_(°C)	Yield (%)
α-1,3-glucan acetate	3	48	16	7.1	2.3	350	177	258	339	73
α-1,3-glucan propionate	3	72	21	8.9	2.3	355	119	239	299	72
α-1,3-glucan from *in vitro* synthesis at 30 °C			20	9.0	2.3	237	—	—	—	12
α-1,3-glucan from *in vitro* synthesis at 15 °C			70	36	1.9	238	—	—	—	1.1

Degree of substitution (DS), number-average (M_n_) and weight-average (M_w_) molecular weights. molecular weight distribution (M_w_/M_n_), temperature at 5% weight-loss (T_d.5%_), glass transition temperature (T_g_), crystallization temperature (T_c_), melting temperature (T_m_) and yield of α-1,3-glucan and its esters (yield (%) = (actual yield/theoretical yield) x 100).
