# Supplementary material for: In vitro synthesis of linear α-1,3-glucan and chemical modification to ester derivatives exhibiting outstanding thermal properties
Source: Sci Rep. 2016 Jul 29;6:30479. doi: 10.1038/srep30479 (PMC4965762; doi:10.1038/srep30479)

Supplementary information

In vitro synthesis of linear α-1,3-glucan and chemical modification to ester derivatives exhibiting outstanding thermal properties

Sakarin Puanglek[1], Satoshi Kimura[1], Yukiko Enomoto-Rogers[1], Taizo Kabe[1,2], Makoto Yoshida[3], Masahisa Wada[4,*], Tadahisa Iwata[1,*]

[1]Department of Biomaterial Sciences, Graduate School of Agricultural and Life Sciences, The University of Tokyo, 1-1-1 Yayoi, Bunkyo-ku, Tokyo 113-8657, Japan

[2]Materials Structure Group 1, Research & Utilization Division, Japan Synchrotron Radiation Research Institute (JASRI), 1-1-1 Kouto, Sayo-cho, Sayo-gun, Hyogo 679-5198, Japan

[3]Department of Environmental and Natural Resource Sciences, Tokyo University of Agriculture and Technology, 3-8-1 Harumi-cho, Fuchu-shi, Tokyo, 183-8509, Japan

[4]Department of Forest and Biomaterials Science, Graduate School of Agriculture, Kyoto University, Kitashirakawa Oiwake-cho, Sakyo-ku, Kyoto 60608502, Japan

*Corresponding authors

E-mail: atiwata@mail.ecc.u-tokyo.ac.jp

Phone: +81-3-5841-5266 Fax: +81-3-5841-1304

Supplementary figures

Figure S1 One-dimensional NMR spectra of α-1,3-glucan acetate. a, ^{1}H-NMR spectrum of α-1,3-glucan acetate. **b,**^{13}C-NMR spectrum of α-1,3-glucan acetate. A sample was prepared in $CDCl_3$.

a

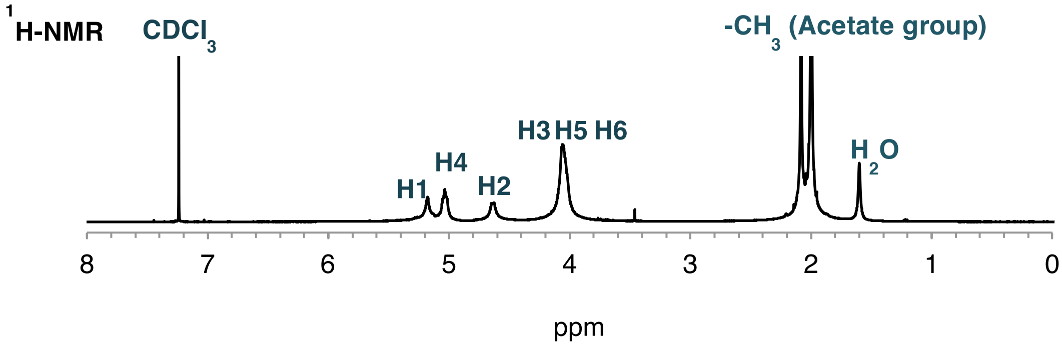

b

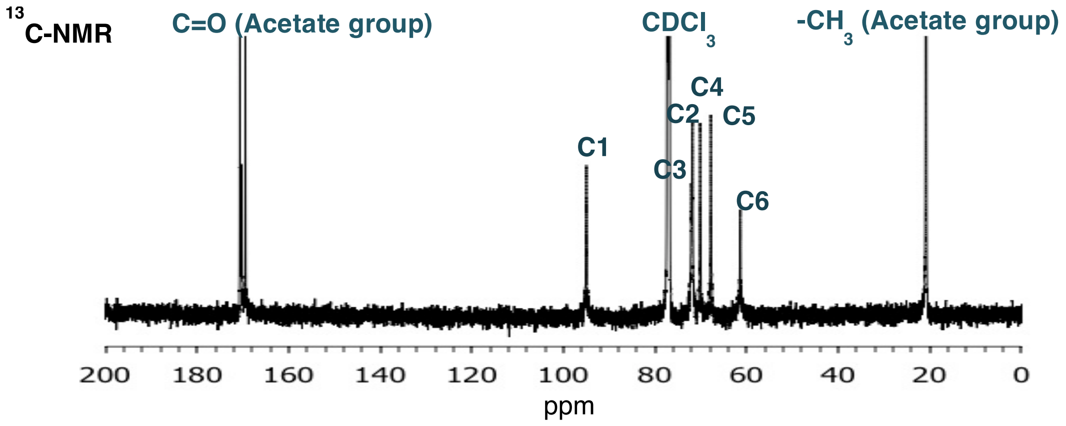

Figure S2 Two-dimensional NMR spectra of α-1,3-glucan acetate. a, HSQC NMR spectrum of α-1,3-glucan acetate. **b,** DQF-COSY NMR spectrum of α-1,3-glucan acetate. A sample was prepared in $CDCl_3$.

a

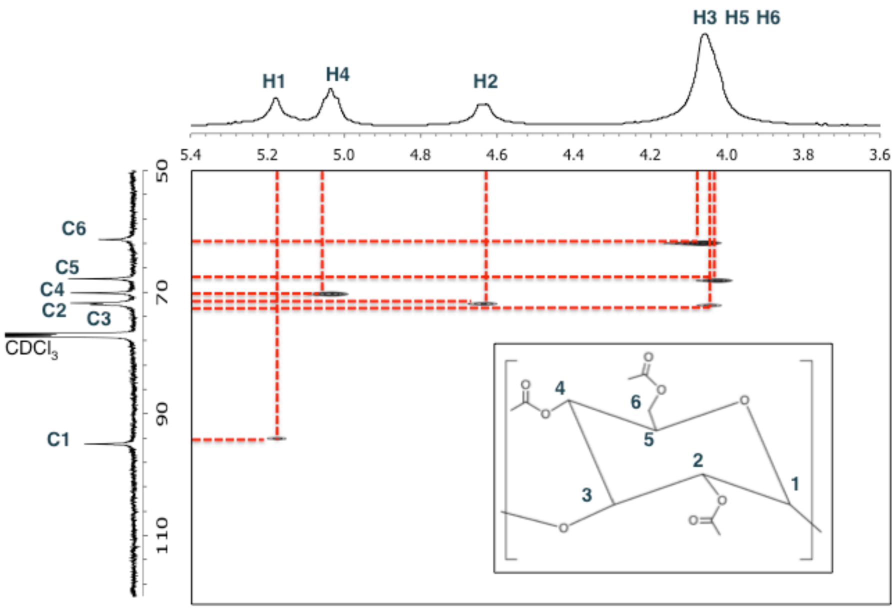

b

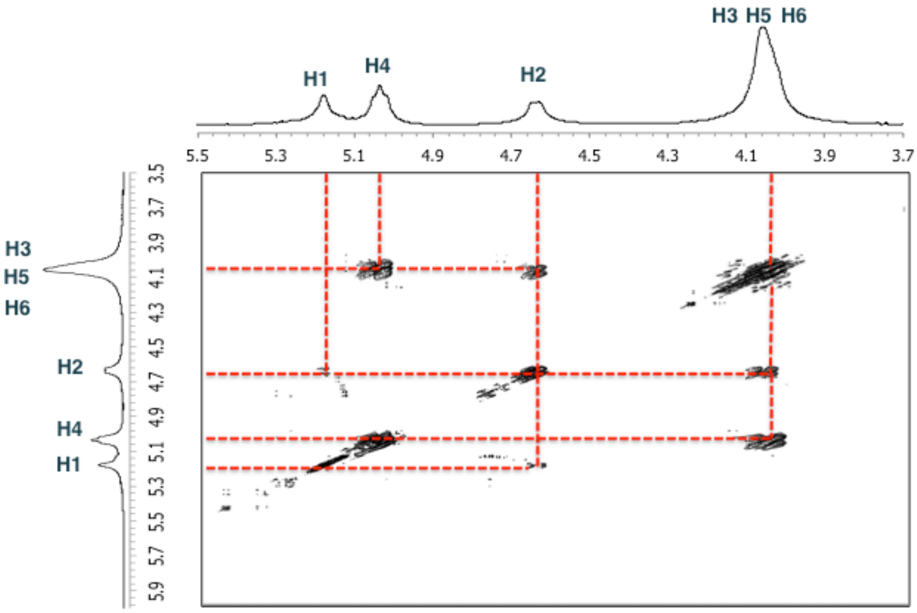

Figure S3 ¹H-NMR spectrum of α-1,3-glucan propionate. A sample was prepared in $CDCl_3$.

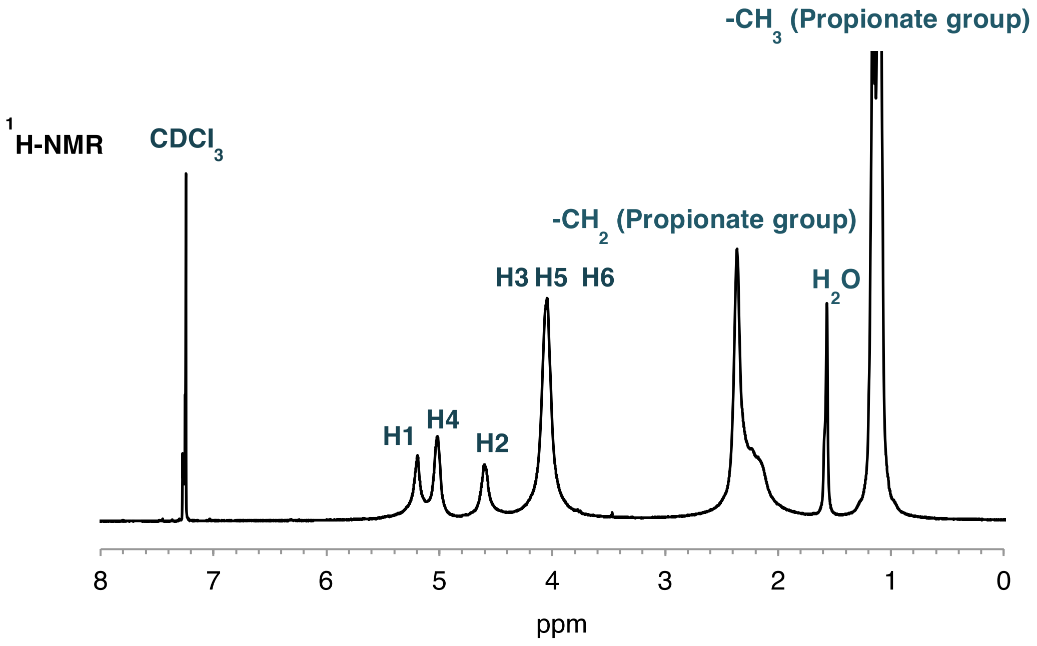

Supplement: Supplementary Information [file srep30479-s1.pdf]
